# Cytometric Evaluation of Cytokine Factors in Serum and Vitreous from Endophthalmitis Patients: Correlated Elevation in Neutrophil Markers

**DOI:** 10.3390/biomedicines13061269

**Published:** 2025-05-22

**Authors:** Christina Carroll, Danica Joseph, Penelope J. Allen, Alex W. Hewitt, Matt Rutar, Rosie C. H. Dawkins

**Affiliations:** 1 Faculty of Science and Technology, University of Canberra, Canberra, ACT 2617, Australia; 2 The Centre for Eye Research Australia (CERA), Melbourne, VIC 3002, Australia; 3 Ophthalmology, Department of Surgery, University of Melbourne, Parkville, VIC 3010, Australia; 4 Vitreoretinal Unit, Royal Victorian Eye and Ear Hospital, East Melbourne, VIC 3002, Australia; 5 Menzies Institute for Medical Research, University of Tasmania, Hobart, TAS 7000, Australia; hewitt.alex@gmail.com; 6 School of Medicine, University of Tasmania, Hobart, TAS 7000, Australia; 7 Department of Anatomy and Physiology, The University of Melbourne, Parkville, VIC 3010, Australia

**Keywords:** endophthalmitis, cytokines, vitreous, neutrophils

## Abstract

**Background**: Endophthalmitis is a rare, sight-threatening condition resulting from infection inside the eye. This study more accurately characterises the cytokines upregulated in human endophthalmitis, and for the first time demonstrates a correlation with cytokine elevation in the serum. **Methods**: We recruited 39 patients, 17 with endophthalmitis and 22 controls. We compared cytokine expression quantified through cytometric bead assays for both vitreous and serum. **Conclusions:** The cytokine profile in the vitreous of patients with infectious endophthalmitis was suggestive of a highly inflammatory environment, as 23/26 cytokines examined were significantly elevated. In the patient sera, MMP-9, MPO, Calprotectin, NGAL, SAA (HVIP1), and MCP-1 (HIP1) were all significantly elevated in endophthalmitis samples, which was unexpected as pathology was thought to be localised with minimal systemic effects. Overall, many of the observed cytokines in endophthalmitis are associated with neutrophil responses, and we believe that this deserves further investigation with a view to developing immunomodulatory therapies to prevent endophthalmitis or improve clinical outcomes. Furthermore, our novel demonstration that cytokine elevation associated with endophthalmitis can be demonstrated in serum may allow for novel and rapid interventions.

## 1. Introduction

Endophthalmitis is a rare, sight-threatening condition resulting from bacterial or fungal infection of the vitreous humour. It may occur secondary to exogenous pathogens introduced to the vitreous milieu from ocular procedures or injury, or less commonly via haematogenous spread from distant sites of infection (2–8%) [1].

The causative pathogen and visual outcome of endophthalmitis vary with aetiology. The adoption of sterile preparation techniques and prophylactic intracameral antibiotics have significantly decreased the incidence of post-procedure endophthalmitis [2,3]. However, with an increasing volume of intraocular surgery and intravitreal injections, these aetiologies do appear more prevalent [3,4].

Though identification of an infectious pathogen may assist in providing adequate antimicrobial treatment, culture-negative endophthalmitis comprises up to 30% of cases and is not associated with worse visual outcome [5,6]. Moreover, it appears that final visual acuity is influenced by the degree of intraocular inflammation and activation of the innate immune response by toll-like receptors (TLRs) [7,8].

Current treatment for bacterial endophthalmitis is limited to intravitreal injection of empirical antibiotics, with the addition of vitrectomy for severe cases. Evidence for the use of steroids to decrease the inflammatory response has remained inconclusive, and the emergence of antimicrobial resistance suggests alternate treatment options need to be considered. By identifying cytokines upregulated in human bacterial endophthalmitis, we provide a potential basis for future targeted immunomodulatory treatments that may better protect against retinal architecture disruption and subsequent vision loss.

## 2. Methods

### 2.1. Selection Criteria

Patients were recruited from the Royal Victorian Eye and Ear Hospital (RVEEH) in two periods, from June 2021 to January 2022, and March 2024 to July 2024. The study was conducted in accordance with the Declaration of Helsinki, with approval from the Royal Victorian Eye and Ear Hospital Human Research Ethics Committee on 5 May 2020 (Protocol 11/988H/21 “*Melbourne Biobank for Eye Disease*”). Control patients were defined as patients undergoing elective surgery for full-thickness macular hole repair (FTMH) with or without combined cataract surgery, and were consented for peri-operative and intra-operative collection of blood and vitreous, respectively.

Patients presenting to RVEEH with endophthalmitis were eligible for inclusion in the case group. All patients presenting with endophthalmitis were eligible to be included in the study. These patients consented for blood collection as well as vitreous collection if undergoing a vitrectomy or if excess vitreous biopsy was obtained during intravitreal tap and injection procedures.

Case group patients were excluded from blood or vitreous sample if it had been longer than 48 h since either initial presentation or intravitreal tap and inject. Patients who had already undergone a vitrectomy prior to development of FTMH or endophthalmitis were excluded from vitreous cavity biopsy. Written informed consent was obtained from all participants.

### 2.2. Sample Collection

Patients undergoing vitrectomy had 0.6–1.0 mL undiluted vitreous collected using a 3 mL syringe attached to the aspiration port. Where the procedure was combined with cataract surgery, cataract extraction was performed prior to obtaining a vitreous sample. Blood was collected via venepuncture in EDTA vacutainers, from BD (Franklin Lakes, NJ, USA). Both blood and vitreous samples were stored on ice from time of collection to time of aliquoting. Fresh undiluted vitreous samples were aliquoted into 0.2 mL samples in 1.5 mL Eppendorf tubes and stored at −80 degrees Celsius until time of processing. To obtain plasma, blood samples were centrifuged at 1300 rpm for 10–15 min at 4 degrees Celsius within two hours of collection. The resulting supernatant was pipetted into 1.5 mL Eppendorf tubes and stored at −80 degrees Celsius. All samples collected were de-identified.

### 2.3. Cytometric Bead Assays

Changes in cytokines and factors between control and IE cohorts were assessed using cytometric bead assays (CBAs), from Biolegend (San Diego, CA, USA); Human Inflammation Panel 1, HIP1 (IL-1β, IFN-α2, IFN-γ, TNF-α, MCP-1, IL-6, IL-8, IL-10, IL-12p70, IL-17A, IL-18, IL-23, IL-33), and Human Vascular Inflammation Panel 1, HVIP1 (Myoglobin, Calprotectin, NGAL, CRP, MMP-2, OPN, MPO, SAA, IGFBP-4, ICAM-1, VCAM-1, MMP-9, and Cystatin C). Both CBA kits were applied according to the manufacturer’s instructions, for both vitreous (*n* = 10–19) and blood (*n* = 15–20) samples, and run on a Northern Lights spectral flow cytometer (Cytek Biosciences, CA, USA).

### 2.4. Data Analysis and Statistics

The raw CBA data were exported to Biolegend’s online Data Analysis Software Suite (version 2024-6-15) for initial processing, before median fluorescence intensity (MFI) values were exported to R (4.4.2) for further analysis. Each LegendPlex panel was analysed separately to account for batch variation. Sample replicates were averaged, and MFIs were log-transformed to equalise variance before being analysed using a mixed effects model with patient as a random effect using the lmerTest package (3.1-3). Pairwise contrasts were then performed using estimated marginal means, and back-transformed from the log data using the emmeans package. Residuals were checked using the ggResidpanel (0.3.0) to ensure the model was appropriate. Values were considered significant where *p* < 0.05. PCA was performed using the FactoMineR (2.11) and FactoExtra (1.0.7) packages and the retained principal components were determined using Scree plots and parallel analysis (paran package (1.5.3)). Correlation plots of significantly elevated cytokines in the vitreous were generated using the corrplot (0.95) package to obtain Pearson correlation coefficients and *p*-values.

## 3. Results

Thirty-nine patients (16 male, 24 female) were enrolled in the study, seventeen in the case group (endophthalmitis) and twenty-two in the control group. The mean age of the control patients was 68 yrs (range 34–82), and 67 yrs (range 12–91) in the case group. Patients in both groups were of predominantly white ethnicity (Table 1).

In the control group (*n* = 22), eleven patients had isolated vitrectomy for FTMH repair and eleven had FTMH repair combined with cataract surgery. Causes of endophthalmitis (Table 2) included preceding intravitreal injection (*n* = 6), cataract surgery (*n* = 5), aqueous paracentesis (*n* = 1), bleb-associated pathologies (*n* = 3), penetrating eye injury (*n* = 1), and endogenous endophthalmitis (*n* = 1).

### 3.1. Cytokine Analysis

**Figure 1:**
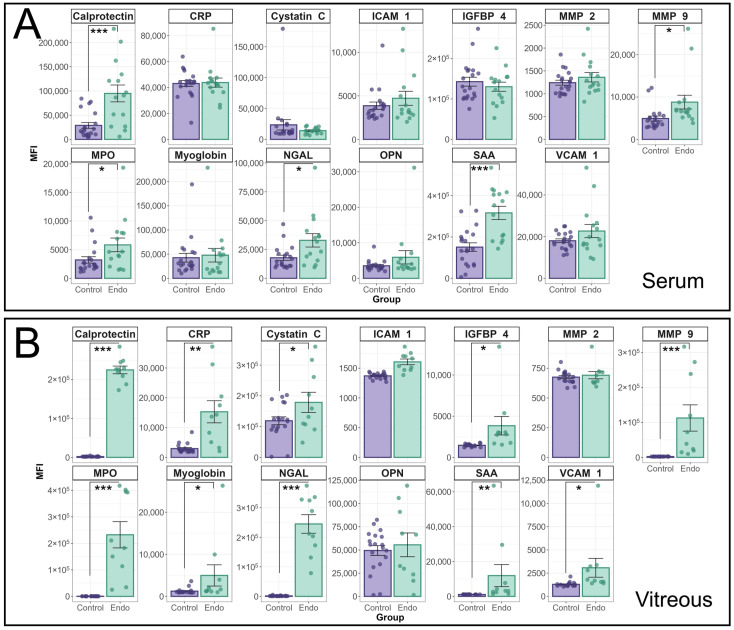
Analysis of cytokine profiles in serum and vitreous from endophthalmitis and control patients using the BioLegend Legendplex Human Vascular Inflammation 1 panel. Serum (**A**) and vitreous (**B**) from patients with endophthalmitis (green) and controls (purple) were tested for the presence of cytokines using the Biolegend LegendPlex cytometric bead assay kit (Human Vascular Inflammation Panel 1). For statistical analysis of the cytokine profiles associated with endophthalmitis, a mixed linear model was used to identify significant differences between groups, with patient included as a random effect. As patient samples were tested in duplicate, the replicates for each patient were averaged, and the Log-transformed MFI values were analysed using the lme4 and lmerTest packages. Residuals were used to confirm the appropriateness of the model (ggResidspanel). *p*-values are representative of the Pr(>F) value. Error bars indicate +/− SE. *p*-values: * = <0.05, ** = <0.001, *** = <0.0001.

The median fluorescence intensity of individual cytokines in the HVIP1 LegendPlex assay between endophthalmitis patients and controls is shown in Figure 1. Cytokine MFIs were analysed using a random effects model and a significant interaction between patient group (i.e., endophthalmitis or control) and cytokine was identified for both serum (*p* < 0.0001) and vitreous (*p* < 0.0001) samples. In patient serum (Figure 1A), significant increases were observed for a number of cytokines, including MMP-9 (*p* = 0.0155); MPO (*p* = 0.0145); Calprotectin (*p* < 0.0001); NGAL (*p* = 0.0144); and SAA (*p* < 0.0001). In the vitreous (Figure 1B), the majority of cytokines tested were significantly elevated in endophthalmitis patient samples compared with control patients, including CRP (*p* = 0.0004); Cystatin C (*p* = 0.048); IGFBP-4 (*p* = 0.017); MMP-9 (*p* < 0.0001); MPO (*p* < 0.0001); Calprotectin (*p* < 0.0001); myoglobin (*p* = 0.0151); NGAL (*p* < 0.0001); SAA (*p* < 0.0001); and VCAM-1 (*p* = 0.0387). There were no significant increases in ICAM-1, MMP2, or OPN in either blood or vitreous.

**Figure 2:**
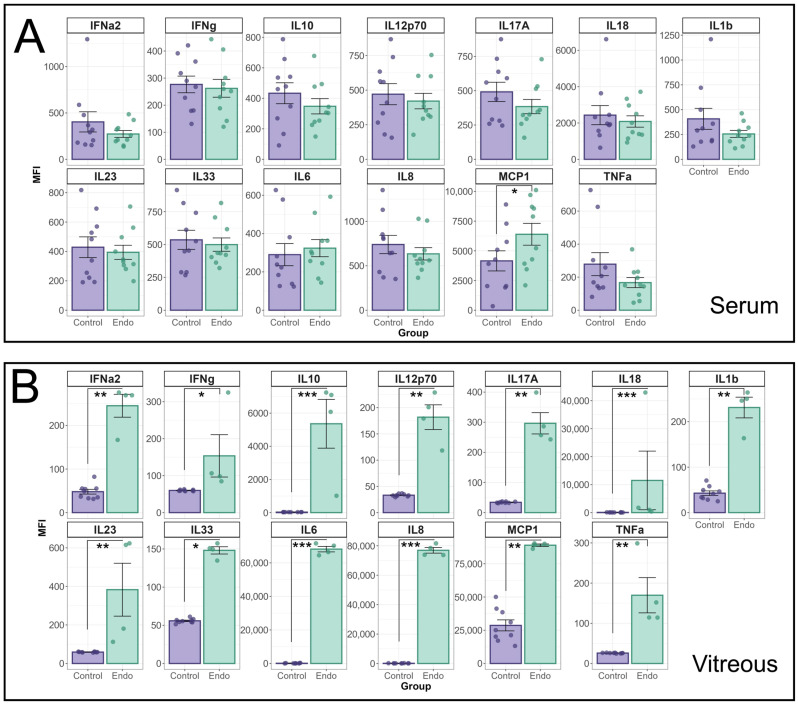
Analysis of cytokine profiles in serum and vitreous from endophthalmitis and control patients using the BioLegend Legendplex Human Inflammation 1 panel. Serum (**A**) and vitreous (**B**) from patients with endophthalmitis (green) and controls (purple) were tested for the presence of cytokines using the Biolegend LegendPlex cytometric bead assay kit (Human Inflammation Panel 1). For statistical analysis of the cytokine profiles associated with endophthalmitis, a mixed linear model was used to identify significant differences between groups, with patient included as a random effect. As patient samples were tested in duplicate, the replicates for each patient were averaged, and the Log-transformed MFI values were analysed using the lme4 and lmerTest packages. Residuals were used to confirm the appropriateness of the model (ggResidspanel). A significant interaction between patient group and cytokine was identified for both serum (*p* = 0.0099) and vitreous (*p* < 0.0001) groups. Estimated marginal means (emmeans package) using pairwise contrasts were calculated and back-transformed. *p*-values are representative of the Pr(>F) value. Error bars indicate +/− SE. *p*-values: * = <0.05, ** = <0.001, *** = <0.0001.

Additional cytokines were analysed using the Biolegend LegendPlex Human inflammation Panel 1 (HIP1) (Figure 2). The MFI of individual cytokines in the HIP1 panel between endophthalmitis patients and controls were compared using a linear mixed effects model (Figure 2A). A significant interaction effect between patient group and cytokine was identified for both serum (*p* = 0.01) and vitreous (*p* < 0.0001) groups. In patient sera, only MCP-1 was found to be significantly elevated (*p* = 0.022) in endophthalmitis samples (Figure 2A). In the vitreous, pairwise contrasts identified significant elevations in all cytokines tested (Figure 2B): IFN-α2 (*p* = 0.0007); IFN-γ (*p* = 0.03); IL-10 (*p* < 0.0001); IL12p70 (*p* = 0.0007); IL-17A (*p* = 0.0002); IL-18 (*p* < 0.0001); IL-1β (*p* = 0.0006); IL-23 (*p* = 0.0008); IL-33 (*p* < 0.0001); IL-8 (*p* < 0.0001); MCP-1 (*p* = 0.0035); and TNF-α (*p* = 0.0005).

### 3.2. PCA

**Figure 3:**
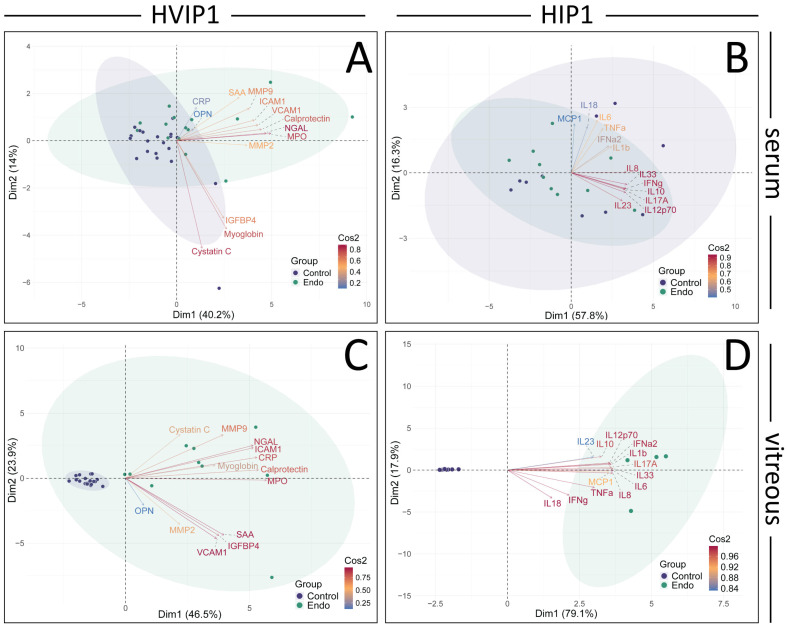
Principal component analysis (PCA) and Pearson’s correlation plots of cytokines in Endophthalmitis samples. PCA was performed on the averaged MFI values for both HVIP1 (**A**,**C**) and HIP1 (**B**,**D**), using the FactoMineR package in R. The number of principal components to retain was determined using Scree plots, eigenvalues, parallel analysis (paran package), and associated adjusted eigenvalues. Two principal components were retained for further analysis. Biplots were generated showing PCA scores for each patient (control: purple, endo: green), and loadings for each cytokine coloured by their cos2 value. Biplots were generated for both serum (**A**,**B**) and vitreous (**C**,**D**) samples, showing PCA scores for each patient (control: purple, endo: green), and loadings for each cytokine coloured by their cos2 value. The proportion of the variance represented by the principal components are shown on the x and y axes.

The cytokine panels were further analysed using PCA and retained principal components validated using parallel analysis (Figure 3). In the HVIP1 serum (Figure 3A), the first two principal components accounted for 54.1% of the variance. NGAL, MPO, and Calprotectin were the cytokines with the greatest impact on the first principal component (PC1), and were therefore the primary contributors to the total variance in the data (contributions 15.60, 14.09, 12.67; and Cos2 values 0.82, 0.76, 0.66, respectively). In HIP1 serum (Figure 3B), 74.1% of the variance could be explained by the first two principal components. IL-33, IL-8, and IL-10 were the greatest contributors to PC1, and the total variance (contributions 14.42, 12.83, 12.52; Cos2 0.87, 0.76, 0.76, respectively). In both cytokine panels, the control and endophthalmitis patient data points overlap, with no clear separation between groups (Figure 3A,B).

**Figure 4:**
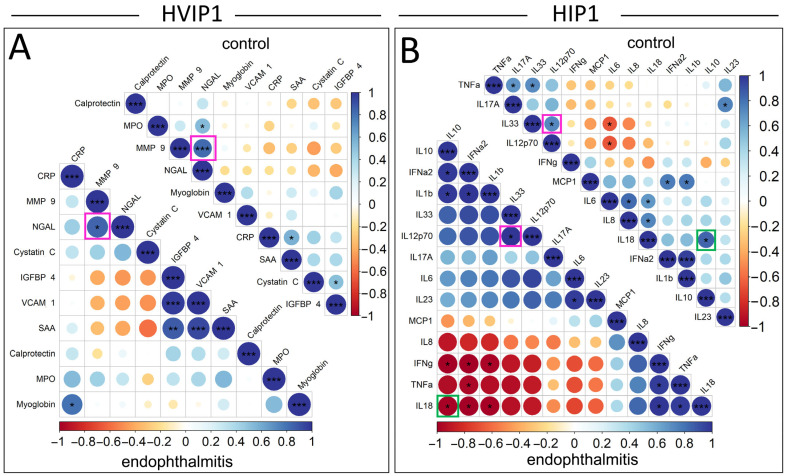
Pearson correlation plots of significantly elevated cytokines in the vitreous. Pearson correlation plots and associated *p*-values were generated using the corrplot package to compare the individual cytokines in a pairwise manner for both the HVIP1 (**A**) and HIP1 (**B**) assays. Cytokines are ordered based on the angular order of eigenvectors, with positive correlations shown in blue, and negative correlations in orange/red. The colour intensity is provided on a scale of 1 to −1, which is proportional to the correlation coefficients. The areas of the circles are proportional to the absolute values of their corresponding correlation coefficients. Pink squares indicate significant pairwise correlations that are consistent between endophthalmitis and control groups. Green squares indicate significant pairwise correlations that occur in both endophthalmitis and control groups, but are opposite in their direction of correlation. *p*-values: * = <0.05, ** = <0.001, *** = <0.0001.

In HVIP1 vitreous samples (Figure 3C), the PCA showed that the first two principal components accounted for 70.4% of the total variance, with MPO, Calprotectin, and CRP having the greatest impact on PC1 (contributions of 0.93, 0.88, 0.87, and Cos2 values of 14.42, 12.83, 12.52, respectively). The control samples are tightly clustered together, while the endophthalmitis group is less tightly clustered on the biplot. In HIP1 vitreous (Figure 3D), 97% of the variance in the data were accounted for by the first two PCs, with the greatest contributors being IL-6, IL-33, and IL-8 (contributions 9.67, 9.59, 9.56; Cos2 0.99, 0.99, 0.98, respectively). The control and endophthalmitis patient groups from both panels form distinct clusters on the biplot (Figure 4B), with the controls grouped more tightly together than the endophthalmitis group, indicating that there is more variation in the endophthalmitis group (Figure 3C,D).

### 3.3. Pearson’s Correlation

The relationships between significantly elevated cytokines in the vitreous samples were analysed using Pearson correlation plots, and compared between endophthalmitis and control groups (Figure 4). Pearson’s correlations were not included for serum samples due to the low number of significantly elevated cytokines. The cytokines in the Pearson’s correlation plots were ordered by the angular order of eigenvectors (AOE), which showed some correlations potentially clustered in pairs or small groups. In the HVIP1 vitreous (Figure 4A), there were a number of significant correlations identified: CRP/Myoglobin, IGFBP-4/SAA, IGFBP-4/VCAM-1, and VCAM-1/SAA in the endophthalmitis group; MPO/NGAL, CRP/SAA, and Cystatin C/Myoglobin in controls; and NGAL/MMP-9 in both groups. All significantly correlated cytokine pairs were found to be positively correlated in the HVIP1 panel.

There were also a number of significantly correlated cytokines in both endophthalmitis and control groups with the HIP1 panel (Figure 4B). In the endophthalmitis group, there were seven positively correlated cytokine pairs, including IL-6/IL-23 and IL-33/IL12p70, which were positively correlated. There were also three potential clusters of cytokines—IFN-α2/IL-10, IL-10/IL-1β, IFN-α2/IL-1β; and IFN-γ/TNF-α, IFN-γ/IL-18, and TNF-α/IL-18—which were all positively correlated. There was also one potential cluster including IFN-γ/IL-10, IFN-γ/IFN-α2, IFN-γ/IL-1β, TNF-α/IFN-α2, IL-18/IL-10, IL-18/IFN-α2, and IL-18/IL-1β, which were all negatively correlated.

Additionally, a number of significant correlations were observed in the control group only. Significant correlations included IL-6/IL-8, IL-6/IL-18, and IL-8/IL-18, which were all positively correlated and appeared to cluster together. The remainder of control group correlations tended to cluster in pairs, although not exclusively. TNF-α/IL-17A, TNF-α/IL-33 and MCP-1/IFN-α2, MCP-1/IL-1β, were all positively correlated, and IL-6/IL-33 and IL-6/IL-12p70 were negatively correlated.

There were two significant correlations that were consistent between groups, IL-33/IL-12p70, and IFN-a/IL-1β, which were positively correlated. Interestingly, IL-18/IL-10 was also significantly correlated in both groups; however, the correlation was negative in the endophthalmitis group and positive in the controls.

## 4. Discussion

The cytokine profiles in serum from patients with endophthalmitis, as well as control patients, were tested using two Biolegend Legendplex CBA kits, which were capable of detecting and quantifying 26 cytokines in total. In the patient sera, MMP-9, MPO, Calprotectin, NGAL, SAA (HVIP1), and MCP-1 (HIP1) were all significantly elevated in endophthalmitis samples (Figure 1 and Figure 2), which was unexpected as pathology was thought to be localised with minimal systemic effects. From the PCA of the sera, the greatest contributors to the total variance in the data were NGAL, MPO, and Calprotectin (Figure 3A), and IL-33, IL-8, and IL-10.

The cytokine profile in the vitreous was suggestive of a highly inflammatory environment, as 23/26 cytokines were significantly elevated, with ICAM-1, OPN, and MMP-2 being the only exceptions. From the PCA (Figure 3), MPO, Calprotectin, IL-33, and IL-8 were among the main contributors to PC1 and therefore the total variance in the data in both the serum and vitreous, while NGAL and IL-10, and CRP and IL-6, also greatly impacted PC1 in the serum and vitreous, respectively.

Due to the limitations of CBAs, it is not possible to discern the cell type responsible for the production of the observed cytokines. However, neutrophils have previously been observed in endophthalmitis animal models [9,10], and a number of the observed cytokines are consistent with those associated with neutrophils. MPO, NGAL, and MMP-9 are major components of azurophillic, specific, and gelatinase granules, respectively, which are deployed via degranulation during inflammatory responses [11]. Elevated plasma calprotectin is an indicator of neutrophil-related inflammation, observed in states of both bacteraemia and chronic inflammation [12,13,14]. Increased plasma calprotectin has also been observed in non-infectious uveitis [15], and elevated vitreous calprotectin has been observed in animal models of experimental autoimmune uveitis and primed mycobacterial uveitis [16]. It is possible that ocular inflammation as seen in endophthalmitis may initiate a systemic neutrophilic response similar to the blood–ocular barrier breakdown seen in uveitis. Calprotectin, which is released into the extracellular space during inflammation, is an endogenous agonist of TLR-4, and amplifies the pro-inflammatory cascade triggered by TLR-4 signalling [17]. TLR-4 is a significant contributor to the intraocular inflammatory response seen in endophthalmitis [18]. Calprotectin is not a neutrophil granule component; however, it does comprise over half of the protein content of the cytosol and is associated with a number of neutrophil responses including migration and NETosis [19]. Neutrophil migration is heavily influenced by IL-8, which is a neutrophil-specific chemotactic molecule that attracts neutrophils to sites of inflammation, as well as inducing their activation [20]. IL-33 is an IL-1 type cytokine that is released extracellularly following cellular damage, where it acts as an alarmin [21] and activator of endothelial cells [22]. IL-33 is cleaved by neutrophil proteases, including NE and Cathespin-G, to produce its bioactive form, which has approximately 10-fold higher activity [23]. IL-33 has demonstrated protective factors in regulating RPE autophagy in macular degeneration [24] and retinal detachment. However, bioactive IL-33 within the vitreous acts as a pro-inflammatory cytokine, and is associated with outer retina disruption and photoreceptor loss when upregulated after retinal injury [25]. SAA, which was significantly increased in both serum and vitreous, is an acute phase protein which acts in the regulation of IL-1β production in neutrophils by activating the NLRP3 inflammasome [26], resulting in respiratory burst [27].

There were a number of cytokines significantly correlated with one another in the vitreous samples, which were largely inconsistent between the endophthalmitis and controls, which may suggest that these cytokines could be co-regulated or involved in common signalling pathways, which are differentially regulated in endophthalmitis. The identification of some significantly correlated cytokines in the control group could be influenced by the fact that in this study these were not true naive controls, as while these patients did not have endophthalmitis, there were clinically relevant reasons for the removal of the vitreous. As such, the presence of some inflammatory markers as a result cannot be ruled out.

The observation that NGAL and MMP-9 were correlated in both endophthalmitis and control vitreous is consistent with previous reports that these cytokines form heterodimers [28]. Vitreous MMP-9 has demonstrated a positive correlation with the severity of diabetic retinopathy [29], with the suggestion that MMP inhibitors may lead to better visual outcomes [30]. The opposite direction of the correlation between IL-10 and IL-18 in endophthalmitis and control groups is interesting, as IL-10 is known to be a regulator of IL-18 signalling, and subsequent IFN-γ induction by NK cells [31]. IL-18 is also known to activate neutrophils and induce respiratory burst and degranulation [32], which is consistent with the neutrophil granule proteins which were identified as being significantly elevated in this study.

Whilst we reached statistical significance with many results, we acknowledge that the relatively small number of specimens is a potential limitation of this study. Additionally, while many of the observed cytokines are associated with neutrophil responses, these cytokines are not exclusively produced by neutrophils, and further investigation is needed to characterise the composition of the immune cell pool contributing to the inflammation induced by endophthalmitis.

## Figures and Tables

**Table 1:** Demographic data of study groups. M = male, F = female.

		Control Group (*n* = 22)	Case Group (*n* = 17)
**Mean age (range) yrs**		68 (34–82)	67 (12–91)
**Sex**		8M (36.4%) 14F (63.6%)	9M (52.9%) 8F (47.1%)
**Ethnicity**			
	White	16 (72.7%)	14 (82.4%)
	Arabic	1 (4.5%)	2 (11.8%)
	East Asian	3 (13.6%)	0
	South Asian	1 (4.5%)	1 (5.9%)
	Pacific Islander	1 (4.5%)	0

**Table 2:** Endophthalmitis samples by aetiology.

	Case Group (*n* = 17)	Vitreous (*n* = 10)	Plasma (*n* = 15)
Post-intravitreal injection	6	3	6
Post-cataract surgery	5	2	5
Bleb-associated	3	3	2
Post-traumatic	1	1	0
Endogenous	1	0	1
Post-aqueous paracentesis	1	1	1

## Data Availability

Underlying data available on request from corresponding author.
